# Effect of Different Luting Methods on the Microtensile Bond Strength of CAD/CAM Resin Blocks

**DOI:** 10.3390/biomimetics10020123

**Published:** 2025-02-19

**Authors:** Alexandra Vinagre, Carla Delgado, Gabriela Almeida, Ana Messias, João Carlos Ramos

**Affiliations:** 1 Institute of Operative Dentistry, Faculty of Medicine, University of Coimbra, 3000-075 Coimbra, Portugal; avinagre@fmed.uc.pt (A.V.);; 2 Center for Innovation and Research in Oral Sciences (CIROS), Faculty of Medicine, University of Coimbra, 3000-075 Coimbra, Portugal; 3 Dentistry Department, Faculty of Medicine, University of Coimbra, 3000-075 Coimbra, Portugalgabriela.almeida00@gmail.com (G.A.); 4 Institute of Prosthodontics and Oral Implantology and, Faculty of Medicine, University of Coimbra, 3000-075 Coimbra, Portugal; 5 Center of Mechanical Engineering Materials and Processes (CEMMPRE), University of Coimbra, 3030-788 Coimbra, Portugal

**Keywords:** CAD/CAM, indirect restorations, film thickness, microtensile bond strength, resin composite

## Abstract

The widespread implementation of new CAD/CAM materials has led to the necessity of establishing an adequate luting protocol. The aim of this study was to evaluate the microtensile bond strength (μTBS) and the film thickness of different luting methods on CAD/CAM resin blocks. Five Brilliant Crios CAD/CAM blocks (Coltene/Whaledent) were sequentially sectioned into two halves, air abraded with 50 µm aluminum oxide, and luted according to five different cementation protocols: Brilliant EverGlow (BEG), Brilliant EverGlow with ultrasound application (BEG-US), preheated Brilliant EverGlow (BEG-H), Brilliant EverGlow Flow (BEGF), and Duo Cem^®^ Trans (DC). Subsequently, the blocks were sectioned to obtain rods, which were then submitted to a microtensile bond strength test (n = 20). The surfaces were examined with optical microscopy to determine the failure mode and the bonding interface was assessed with scanning electron microscope (SEM) analysis. Bond strength values were analyzed using one-way ANOVA and Tukey’s post hoc tests (α = 0.05). The bond strength values varied with the different cementation protocols (*p* < 0.001): BEG (45.48 ± 18.14 MPa), BEG-US (42.15 ± 14.90 MPa), BEG-H (41.23 ± 15.15 MPa), BEGF (58.38 ± 15.65 MPa), and DC (81.07 ± 8.75 MPa). Regarding bond strength, DC presented significantly higher values than all other experimental groups (*p* < 0.050), whereas all luting methods using BEG presented similar values (*p* = 0.894). Adhesive failures were the predominant type. On SEM evaluation, all the luting materials presented a tight and homogeneous cement–block interface with variable film thicknesses. In conclusion, among the cementation protocols, the resin cement (DC) rendered the highest bond strength values. SEM analysis revealed that the lowest film thickness was associated with the flowable composite (BEGF).

## 1. Introduction

Computer-aided designed and computer-aided manufactured (CAD/CAM) composites are among the most studied materials in restorative dentistry, competing with glass ceramics for single-unit indirect restorations [1]. Digital systems enable restorative procedures to be completed in a single appointment, as they allow digital impressions, restoration design, and chairside milling of CAD/CAM blocks, which can then be bonded almost immediately [2,3]. The use of these indirect techniques is recommended for large cavities, typically involving one or more cusps and proximal surfaces, consequently requiring restorations with superior mechanical properties [2].

Composite blocks can be classified based on their microstructure, according to the incorporation of dimethacrylates, such as urethane dimethacrylate (UDMA) and triethylene glycol dimethacrylate (TEGMA), into the matrix. While dispersed fillers are mixed and polymerized under high temperature, polymer-infiltrated ceramic network materials are secondarily infiltrated and polymerized under high temperature and high pressure [4,5].

CAD/CAM composite blocks exhibit a higher degree of conversion, addressing some disadvantages of direct restorations by minimizing flaws and pores, thus enhancing material homogeneity [1,5,6,7]. Additionally, studies consistently report that resin composite blocks exhibit higher flexural strength, flexural modulus, and microhardness compared to direct composite resins [8,9,10]. When compared to ceramics, resin composite blocks demonstrate higher resilience, lower elastic modulus, and a more efficient milling process, which is easier, less expensive, and results in reduced marginal chipping, allowing for thinner margins to be milled [1,5,6].

While the high conversion rate of resin composite blocks mostly acts as an advantage, it concomitantly reduces the potential for chemical bonding interactions due to the decreased availability of free carbon double bonds [1,5,6,7]. Consequently, appropriate surface pretreatment of the restoration is essential, with studies identifying air abrasion as the most effective method for increasing surface roughness. This process promotes enhanced micromechanical adhesion to both the adhesive and the luting agent.

Luting is a crucial step in ensuring retention, marginal sealing, and longevity of indirect restorations [11]. Without proper adhesion, restoration failure may occur, leading to microleakage, secondary caries, and pulp inflammation [2,6,7,12]. Therefore, an ideal luting agent must have specific biological, physical, and mechanical properties, providing stable bonding between the remaining dental structure and the restoration, sufficient mechanical strength to withstand masticatory forces, resistance to wear, low solubility in oral fluids, low film thickness, biocompatibility with oral tissues, radiopacity, color stability, and ease of handling [13].

It is noteworthy that luting procedures depend on the restoration material, adhesive system, and luting agent [14]. Furthermore, adhesion to the resin matrix can be achieved through physical, mechanical, or chemical processes [15,16]. While physical adhesion relies on van der Waals forces or hydrogen bonds, requiring resin primers to contain hydroxyl or amino groups that interact with corresponding groups within the matrix, mechanical adhesion occurs when resin primer monomers penetrate the matrix and undergo in situ polymerization. In contrast, chemical adhesion involves the formation of new covalent bonds between monomers and the available double bonds on the substrate [15,16].

In recent years, resin-based cements and composite resins have been the most commonly used luting agents. Resin luting agents are classified by their polymerization mechanisms into light-cured, chemically cured, and dual-cured cements [6]. Composite resins need to undergo rheological modifications to function as luting agents, which can be achieved through ultrasonic vibration or thermal modification to ensure adequate film thickness [17]. Thermally modified composites have shown favorable physical and mechanical properties, though the technique is highly sensitive due to rapid resin cooling upon removal from the heating device [18]. Ultrasonically vibrated composite resins provide high clinical applicability by ensuring more precise restoration adaptation and less technical sensitivity [19].

The luting process of indirect restorations is a critical factor influencing their clinical performance and longevity [15], hence the bonding protocol must be strictly followed [20]. In opposition to the existing extensive research on bonding strategies for ceramics, the available literature lacks studies focusing specifically on the optimal luting protocols for composite resin blocks, providing some valuable insights to enhance clinical longevity. The evolving landscape of adhesive dentistry and the increasing use of CAD/CAM composite restorations requires further investigation on this topic to ensure more predictable clinical outcomes. Thus, the aim of this study was to evaluate the microtensile bond strength (μTBS) and bonding interface of five different luting systems applied to a CAD/CAM composite resin block, Brilliant Crios^®^ (Coltene/Whaledent, Langenau, Germany). Two null hypotheses were tested: (H0a) there are no differences in bond strength among the different luting methods, and (H0b) there are no differences in the micromorphology of the bonding interface produced by different luting methods.

## 2. Materials and Methods

### 2.1. Sample Preparation

Five composite resin blocks, Brilliant Crios^®^ (Coltene/Whaledent, Langenau, Germany, LOT H00414), were transversely sectioned in half with a diamond disk-cutting machine (Accutom 5, Struers, Ballerup, Denmark), at 1000 rpm, with a forward speed of 0.100 mm/s. After the cutting of the block in two halves, both surfaces were sequentially polished, in a circular motion, with #320- and #600-grit silicon carbide (SiC) abrasive papers (WSFlex 16^®^, Hermes Schleifmittel GmbH, Hamburg, Germany), for 60 s, under constant water irrigation. Following the polishing procedure, the two mating surfaces were air abraded with 50 μm aluminum oxide particles (Airsonic^®^ mini sandblaster, Hager Werken, Duisburg, Germany), projected at a 45° inclination. The air abrasion device was positioned 10 mm away from the surface. This distance was standardized by attaching a gutta-percha cone to the tip of the device and the surfaces were air abraded with the device in constant motion, ensuring a total of six linear applications per surface, with no repetitions. Following this, a cleaning protocol was applied in which samples were washed with distilled water, ultrasonically vibrated (BioSonic^®^ UC 125, Coltene, LOT 996P067) in 96% alcohol, for 2 min, and dried with absorbent paper.

The samples were then divided into five groups, according to the tested luting protocols. Experimental groups are depicted in Table 1. The adhesive system (One Coat 7 Universal^®^, Coltene/Whaledent, Langenau, Germany, LOT H14305), employed in all groups, was actively applied with a microbrush for 20 s and air dried for 5 s to promote solvent evaporation. Luting materials were homogeneously distributed on each block surface.

For all luting procedures, a calibrated seating load of 23 Newtons was applied by a patented device [17]. For Groups 1, 2, and 3, Brilliant EverGlow^®^ A2/B2 (Coltene/Whaledent, Langenau, Germany, LOT H15193) was used as the luting agent. In Group 2, the composite resin was associated with ultrasonic vibration (Dentsurg Pro^®^, CVDentus, São José dos Campos, Brazil), with an ultrasonic tip operating at 30% power, without irrigation, and performing smooth movements from the center to the periphery of the specimens. In Group 3, the resin was preheated with a heating unit (Ease-it™ Heating Unit, Ronvig, Daugård, Denmark), at 60 °C. For all experimental groups with composite resin as luting agent, excess removal was performed every 15 s with an OptraSculpt (Ivoclar Vivadent, Schaan, Liechtenstein), ensuring optimal composite overflow. In Group 4, the luting agent was Brilliant EverGlow^®^ Flow (Coltene/Whaledent, Langenau, Germany, LOT H33890). Finally, in Group 5, the adhesive (One Coat 7 Universal) was mixed with the activator Blend (One Coat 7.0 activator^®^, Coltene/Whaledent, Langenau, Germany, LOT H13425), 30 s prior to its application. The dual-cured resin cement DuoCem (Coltene/Whaledent, Langenau, Germany, LOT H01432) was then applied, and the luting interfaces were light cured (Bluephase Style 20i^®^, Ivoclar Vivadent, Lichenstein, 1200 mW/cm^2^) for 20 s, four times, for a total of 80 s.

The blocks were stored in distilled water at 37 °C, for 24 h, prior to microspecimen preparation. All the materials, manufacturers, LOT, and compositions are displayed in Table 2. 

### 2.2. Microtensile Bond Strength Test (µTBS)

After the cementation procedures, each block was sectioned with a precision cutting machine (Accutom 5, Struers, Ballerup, Denmark), at a slow speed of 1000 rpm, at 0.100 mm/s, under permanent water cooling. The blocks were cut parallel to their long axis and perpendicular to the adhesive interface then rotated 90º degrees along the same axis to be sectioned again. The peripheral rods of each block were excluded, as well as those presenting any defect or void within the adhesive interface, resulting in a total of twenty rods per study group. Then, the cross-sectional area of all rods (mm^2^) was measured using a digital caliper (Mitutoyo digital caliper, Japan), with the average adhesive area being approximately 0.9 mm^2^.

**Figure 1:**
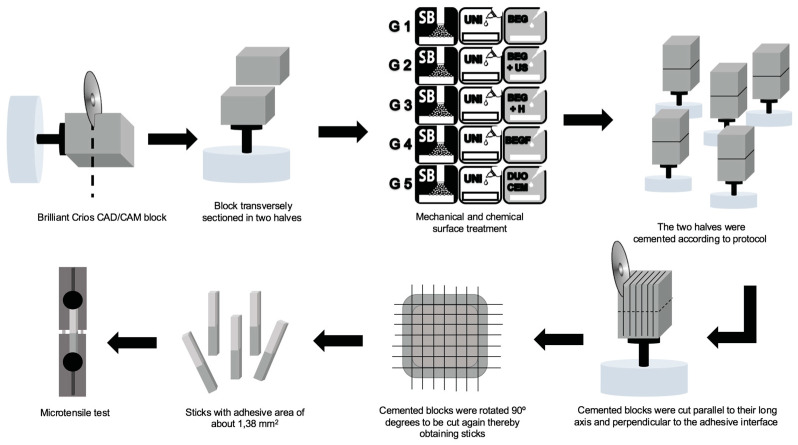
Schematic representation of the experimental procedure. Abbreviations: SB: Sandblasting with 50 μm Al2O3; UNI: Universal adhesive.

Microtensile bond strength (µTBS) testing was performed using a highly precise universal testing machine (Autograph^®^, Model AG-I, Shimadzu Corporation, Kyoto, Japan) to evaluate the adhesive performance of the bonded specimens. During the testing process, the resin composite rods were subjected to an increasing tensile force until failure, applied at a controlled rate of 0.5 mm/min to ensure consistent stress distribution. For each individual rod, the exact load at failure was recorded in Newtons (N) and subsequently divided by the previously measured cross-sectional area (mm^2^) to accurately determine the microtensile bond strength (MPa). This method allowed for precise quantification of the adhesive properties of different bonding protocols. Figure 1 displays a schematic representation of the experimental procedure.

### 2.3. Failure Mode Analysis

The failure mode was analyzed under an optical microscope (Leica M300, Leica Microsystems, Heerbrugg, Switzerland) with a ×35 magnification. The fracture pattern was classified as follows: (A) adhesive at the bonding interface, (CC) cohesive in the CAD/CAM block, (CL) cohesive in the luting agent, and (M) mixed, both cohesive in the luting agent and in the CAD/CAM block.

### 2.4. Scanning Electron Microscopy (SEM)

Adhesive interface evaluation of each group was conducted with a scanning electron microscope (SEM). Two additional samples from each group were polished using abrasive sandpaper with an ascending grit series (500, 1000, 1200, and 2500 grit, respectively).

The specimens were then rinsed with an ascending series of ethanol (50, 75, 90, and 100%) for 15 min per solution and further sonicated in absolute ethanol for the same time to complete dehydration. All samples were positioned in aluminum supports and sputter coated with gold–palladium (Polaron E-5000 Sputter-Coater, Polaron Equipment Lta, Watford, UK) for further observation on a scanning electron microscope (Hitachi S-4100 microscope; Hitachi, Tokyo, Japan) with an accelerating voltage of 25 kV, at ×250 and ×2500 magnifications.

### 2.5. Statistical Analysis

Statistical analysis was performed with the IBM SPSS Statistics 23.0^®^ program (SPSS Inc., Chicago, IL, USA). One-way analysis of variance (ANOVA) was used to compare means of microtensile bond strength data between groups with Tukey post hoc pairwise comparisons. The chi-square test was used to determine the independence of group and failure mode variables. The significance level was set at α = 0.05.

## 3. Results

### 3.1. Microtensile Bond Strength

**Figure 2:**
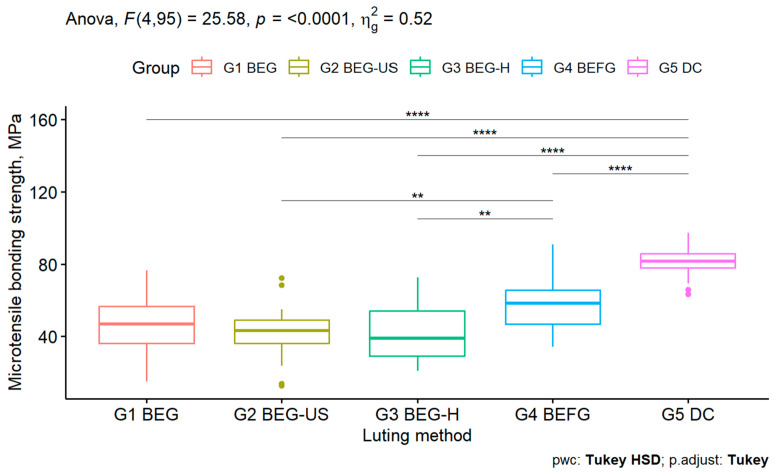
Schematic representation of µTBS results by group with corresponding statistical analysis (ANOVA and Tukey pairwise comparisons). Lines indicate statistically significant differences between pairs of groups. ** *p* < 0.01; **** *p* < 0.0001.

Descriptive statistics, the number of tested specimens, and µTBS results are depicted in Table 3 and schematically represented in Figure 2. The assumptions of normality and homogeneity of variances of the ANOVA were met, with non-significant results for both the Shapiro–Wilk test for each group (*p* > 0.05) and for the Levene test (*p* > 0.05). One-way ANOVA revealed statistically significant differences amongst groups (F(4.95) = 25.6, *p* < 0.01). Pairwise comparisons between groups indicated significant differences among the μTBS mean values of DC (Group 5) and the other groups, the former having the highest bond strength values. Also, BEGF (Group 4) was statistically different from all except for BEG (Group 1). Tukey’s homogenous means subsets are displayed in Table 4. Three different subsets are identified, corresponding to the lowest values of mean μTBS, intermediate values of mean μTBS, and highest values of mean μTBS. All BEG-based groups are within the lowest subset. G1-BEG also shares the intermediate subset with G4-BEGF while G5-DC stands isolated in the highest mean μTBS values subset.

The failure mode frequency and distribution can be analyzed in Table 5. For all groups, failure was predominantly adhesive. No statistically significant associations could be found between group and failure mode (χ^2^(12) = 10.4, *p* = 0.576).

### 3.2. Scanning Electron Microscopy Results

**Figure 3:**
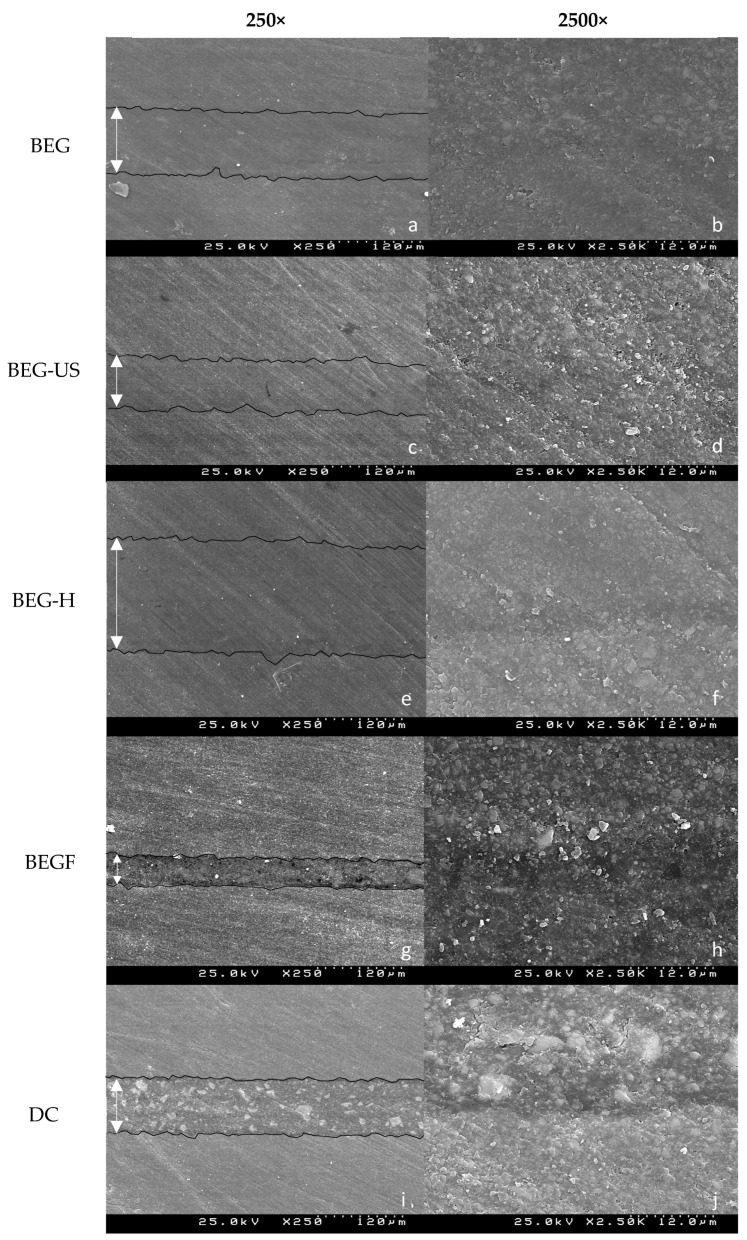
SEM images of the bonding interfaces where arrows indicate cement layer thickness. (**a**) BEG, 250×; (**b**) BEG, 2500×; (**c**) BEG-US, 250×; (**d**) BEG-US, 2500×; (**e**) BEG-H, 250×; (**f**) BEG-H, 2500×; (**g**) BEGF, 250×; (**h**) BEGF, 2500×; (**i**) DC, 250×; (**j**) DC, 2500×.

The photomicrographs obtained by SEM with a beam acceleration of 25.0 kV, at 250× and 2500× magnifications, were qualitatively analyzed. A representative photomicrograph of each group can be seen in Figure 3.

The analysis of the bonding interface demonstrated a well-adapted and tightly sealed interface between the cement and the resin composite block across all tested luting materials. Among the different groups, the BEGF group exhibited the thinnest cementation line. In contrast, the DC and BEG-US groups displayed an intermediate cement layer thickness, while the BEG and BEG-H groups showed a noticeably larger film thickness. Notably, when ultrasonic application was introduced during the cementation process, the resulting cement layer appeared more densely packed, with fewer pores and voids.

## 4. Discussion

The null hypotheses, (H0a) there are no differences in the bond strength among the different luting agents and (H0b) there are no differences in the micromorphology of the bonding interface produced by the different luting agents, were both rejected by the findings of this study, as distinct outcomes were observed for each luting material, regarding both bond strength evaluation and ultrastructural analysis through SEM.

CAD/CAM composite blocks have become a valuable material in dentistry for fabricating precise, aesthetic, and durable restorations, including inlays, onlays, crowns, and veneers [3]. Achieving proper luting plays a pivotal role in the restoration’s clinical success. Therefore, several luting agents and cementation techniques have been studied to understand those rendering superior results, according to bond strength and the obtained film thickness, enabling the clinician to enhance treatment predictability and long-term clinical outcomes [3,17].

Previous studies focused on the bonding properties of CAD/CAM composite blocks, evaluating either shear, microshear, or microtensile bond strength. This study demonstrated superior microtensile bond strength values in comparison to other literature results assessing composite blocks, possibly due to the higher concentration of carbon–carbon double bonds on the surface of the Brilliant Crios CAD/CAM block [7]. Additionally, the fact that all the materials are from the same manufacturer should also be taken into account, with higher chemical compatibility achieved. Although the present study focuses on the bonding interactions within the same resin composite block, it is noteworthy that proper bonding performance should be established on dental tissues, such as enamel or dentin, creating a seamless interface between the restoration and the natural tooth structure. Hence, mechanical properties of the restoration are potentiated, microleakage is minimized, and aesthetic and functional requirements of composite resin restorations are fulfilled [21].

Successful adhesion of indirect restorations can be attained by creating a reliable bond between the internal surface of the CAD/CAM restoration and the luting agent. Therefore, several studies have demonstrated the benefits of air abrasion followed by silanization to enhance micromechanical and chemical interactions [3,22,23]. The International Academy for Adhesive Dentistry recommends resin composite blocks to be pretreated with air abrasion with either 50 µm aluminum oxide or 30 µm silicon oxide particles, at a pressure of 2 bar (0.2 MPa) [4]. This procedure not only increases micromechanical retention but also removes the potential smear layer created by grinding or milling procedures [4]. Tekçe et al. reported that bond strength was higher for specimens air abraded with 50 μm aluminum oxide than for those pretreated with 30 μm silicon dioxide or 27 μm aluminum oxide, for all the composite blocks studied [24]. Furthermore, Yoshihara et al. evaluated the effects of air abrasion on different CAD/CAM blocks with 50 µm aluminum oxide, at 2 bar, concluding that although the procedure was necessary to improve bond strength, microfractures of 1 to 10 μm in the surface of the composite block were observed [22]. Therefore, pressure should not be increased to avoid the possibility of subsurface crack formation [25].

Multiple studies have investigated the effects of various pretreatment methods, including the application of silane coupling agents and different types of resin primers, on a wide range of CAD/CAM resin blocks. These studies collectively concluded that, among the tested pretreatments, the use of a resin primer containing methyl methacrylate (MMA) led to significantly higher bond strength values compared to other conditioning agents [7,26,27]. Hence, for this study, air abrasion pretreatment and the One Coat Universal adhesive system, containing MMA, were applied to all the specimens throughout the experimental groups.

In addition to the surface pretreatment, other factors can interfere with the adhesive cementation of indirect restorations, such as the chosen luting agent. In the literature, resin cements are described as having a high elasticity modulus, high resistance to compression, and satisfactory bond strength [28]. Gilbert et al. demonstrated higher bond strength values for conventional dual-cured resin cements in comparison to a self-adhesive dual-cured resin cement, due to the presence of multifunctional dimethacrylates that allow a substantial chemical bond to PMMA-based CAD/CAM composites [12]. This is also supported by our study, as statistically superior bond strength values were found when the dual-cured resin cement was applied.

Regarding the comparison between BEGF and BEG, statistically significant differences with respect to bond strength were not found among these two groups. In accordance with our study, Lise et al. concluded that the microtensile bond strengths of the specimens luted with conventional or flowable composite were statistically similar [29].

Among the BEG groups, a higher dispersion of the data was obtained due to the higher technical sensitivity of the tested protocols. Both ultrasonic vibration and the preheating pretreatment resulted in statistically similar results to the non-treated composite resin. Regarding the ultrasonic vibration, Cantoro et al. also found that this pretreatment did not have a statistically significant effect on microtensile bond strength [19]. On the other hand, Silva et al. concluded that the ultrasound application increased the bonding performance [30].

Furthermore, the process of preheating the composite resin leads to a reduction in the material viscosity, thereby improving the restoration placement. The systematic review and meta-analysis conducted by Jardim Barbon et al. was in accordance with the present results, as a significant difference in bond strength values could not be found [31]. Contrarily, Pappachini et al. stated that the bond strength improved by increasing the temperature [32]. Additionally, Foes-Salgado et al. reported a significant improvement in marginal adaptation when preheated composites were used as luting agents [33].

Regarding the failure mode, adhesive failures were predominantly found in our study. Cohesive failures reached a 45% prevalence in the BEGF group, which can be explained by the lower flexural strength presented by this material [29].

Another important factor to consider is the thickness of the luting agent layer. According to ISO 4049:2019 [34], an ideal film thickness should be between 5 and 25 µm, never exceeding 50 µm. May et al. reported that failure load decreases as the thickness of the resin cement increases, meaning that the thickness must be the minimum possible in order not to interfere with the mechanical properties of indirect restorations [35].

Ultrastructural analysis through SEM revealed that resin composite blocks are associated with a homogeneous microstructure with well-distributed filler particles, contributing to their enhanced mechanical properties and wear resistance. Although the SEM images of the bonding interface revealed a tight cement–block interface for all the luting materials, the BEGF group was associated with a thinner cementation line, followed by DC. This result can be explained by the composition of Brilliant EverGlow Flow, as these materials are formed by suspending solid ceramic particles in a resin matrix, resulting in viscoplastic fluids, which have the ability to flow easily [36]. Furthermore, Duo Cem^®^ has a considerably lower viscosity compared to conventional composites, at room temperature, due to the lower inorganic filler composition [11].

It is noteworthy that the ultrasonic vibration, in BEG-US, resulted in lower film thickness than in the BEG group, similar to the findings reported by Falacho et al. [17]. Furthermore, images from the BEG-US group display a more densely packed and less porous cement layer. On the other hand, the BEG-H group resulted in the highest film thickness, possibly due to the higher technique sensitivity, as composite temperature decreases 50% within 2 min and 90% in the first 5 min after being removed from the heating unit, which compromises the viscosity and, consequently, the restoration’s marginal adaptation [18,37]. It is important to note that, although preheated composite resin has been associated with reduced film thickness in previous studies which could stand as an advantage [17], other authors reported similar flexural strength values for CAD/CAM composite blocks luted either with preheated composite or with a conventional dual-cure resin cement [38].

It should be emphasized that in vitro studies are unable to simulate all the individual conditions to which a restoration is exposed in the oral cavity, which poses as a limitation for the present study. Additionally, although in our study only immediate bond strength was evaluated, it is imperative to assess the bonding performance of aged specimens to accurately understand the long-term differences between luting agents, as previous studies demonstrate the associated deleterious effects [27]. Furthermore, it is important to draw attention to the fact that the findings of this study cannot be generalized to all CAD/CAM resin composite blocks, despite the fact some authors argue that the effectiveness of the bonding process is mainly dependent on the luting method, rather than on the type of resin composite block [39].

## 5. Conclusions

In conclusion, the bond strength associated with Brilliant Crios CAD/CAM blocks is directly dependent on the employed luting agent. Among the cementation protocols, the resin cement Duo Cem^®^ rendered the highest bond strength value and SEM analysis revealed that the lowest film thickness was associated with the flowable composite Brilliant EverGlow^®^ Flow.

## Figures and Tables

**Table 1:** Experimental groups.

Groups	Protocols
G1-BEG	One Coat 7 Universal^®^ + Brilliant EverGlow^®^ A2/B2
G2-BEG-US	One Coat 7 Universal^®^ + Brilliant EverGlow^®^ A2/B2 with ultrasonic vibration
G3-BEG-H	One Coat 7 Universal^®^ + Brilliant EverGlow^®^ A2/B2 preheated
G4-BEGF	One Coat 7 Universal^®^ + Brilliant EverGlow^®^ Flow A3/D3
G5-DC	One Coat 7 Universal^®^ + One Coat 7.0 activator^®^ + DuoCem^®^ Trans

**Table 2:** Material and product specifications.

Material	Manufacturer (LOT)	Composition
Brilliant Crios^®^ CAD/CAM	Coltene/Whaledent, Langenau, Germany (H00414)	Cross-linked methacrylates (Bis-GMA, BIS-EMA, TEGMA), 71 wt. % barium glass and silica particles
Brilliant EverGlow^®^ (BEG) A2/B2	Coltene/Whaledent, Langenau, Germany (H15193)	Bis-GMA, TEGDMA, Bis EMA, prepolymerized particles containing glass and nanosilica, aggregated and non-aggregated colloidal silica, and barium glass (74 wt. %)
Brilliant EverGlow^®^ Flow (BEGF) A3/D3	Coltene/Whaledent, Langenau, Germany (H33890)	Methacrylates, barium glass, silanized amorphous hydrophobic silica (37 wt. %)
Duo Cem^®^ Sample Trans	Coltene/Whaledent, Langenau, Germany (H01432)	Bis-EMA, Bis-GMA, TEGMA, salinized barium glass, amorphous silicic (60–70 wt. %)
One Coat 7.0 activator^®^	Coltene/Whaledent, Langenau, Germany (H13425)	Bis-GMA, TEGMA, UDMA, fluoride, barium glass, amorphous silicic (68 wt%, 0.1–5 mm), ethanol, water, activator
One Coat 7 Universal^®^	Coltene/Whaledent, Langenau, Germany (H14305)	HEMA, MMA-modified polyacrylic acid, UDMA, amorphous silicic, 10-MDP, ethanol, water, pH = 2.8

Abbreviations: TEGDMA: triethylenglycol dimethacrylate; Bis-EMA: ethoxylated bisphenol-A-diglycidyl methacrylate; UDMA: urethane dimethacrylate; 10-MDP: 10-methacryloyloxydecyl dihydrogen phosphate; HEMA: 2-hydroxyethyl methacrylate; Bis-GMA: bisphenol-A-diglycidyl methacrylate.

**Table 3:** Descriptive statistics of the five groups tested.

Group	n	Mean (MPa)	Std. Deviation	ANOVA
G1-BEG	20	45.48 a,b	18.14	<0.001
G2-BEG-US	20	42.15 a	14.90	
G3-BEG-H	20	41.23 a	15.15	
G4-BEGF	20	58.38 b	15.65	
G5-DC	20	81.07 c	8.75	

Different letters indicate statistically significant differences between the study groups (*p* < 0.05). n = number of samples.

**Table 4:** Tukey HSD homogenous subsets of mean μTBS (MPa) for alpha = 0.05.

Group	Subset 1	Subset 2	Subset 3
G3-BEG-H	41.23		
G2-BEG-US	42.15		
G1-BEG	45.48	45.48	
G4-BEGF		58.38	
G5-DC			81.07
*p*-value	0.894	0.054	<0.050

**Table 5:** Distribution of fracture patterns by groups. Absolute number of samples (%).

	Group	G1-BEG	G2-BEG-US	G3-BEG-H	G4-BEGF	G5-DC
Failure Mode						
Adhesive (A)		16 (80)	15 (75)	14 (70)	11 (55)	17 (85)
Mixed (M)		0 (0)	1 (5)	2 (10)	2 (10)	1 (5)
Cohesive CAD/CAM (CC)		0 (0)	2 (10)	2 (10)	2 (10)	0 (0)
Cohesive luting agent (CL)		4 (20)	2 (10)	2 (10)	5 (25)	2 (10)

## Data Availability

The data presented in this study are available on request from the corresponding author.
